# Sildenafil (Viagra) Protective Effects on Neuroinflammation: The Role of iNOS/NO System in an Inflammatory Demyelination Model

**DOI:** 10.1155/2013/321460

**Published:** 2013-07-22

**Authors:** Catarina Raposo, Ana Karolina de Santana Nunes, Rayana Leal de Almeida Luna, Shyrlene Meiry da Rocha Araújo, Maria Alice da Cruz-Höfling, Christina Alves Peixoto

**Affiliations:** ^1^Departamento de Histologia e Embriologia, Instituto de Biologia, Universidade Estadual de Campinas (UNICAMP), Rua Monteiro Lobato 255, 13083-862 Campinas, SP, Brazil; ^2^Laboratório de Ultraestrutura, Centro de Pesquisas Aggeu Magalhães, Fundação Oswaldo Cruz, Av. Professor Moraes Rego, s/n, Cidade Universitária, 50670-420 Recife, PE, Brazil

## Abstract

We recently demonstrated that sildenafil reduces the expression of cytokines, COX-2, and GFAP in a demyelinating model induced in wild-type (WT) mice. Herein, the understandings of the neuroprotective effect of sildenafil and the mediation of iNOS/NO system on inflammatory demyelination induced by cuprizone were investigated. The cerebella of iNOS^−/−^ mice were examined after four weeks of treatment with cuprizone alone or combined with sildenafil. Cuprizone increased GFAP, Iba-1, TNF-**α**, COX-2, IL-1**β**, and IFN-**γ** expression, decreased expression of glutathione S-transferase pi (GSTpi), and damaged myelin in iNOS^−/−^ mice. Sildenafil reduced Iba-1, IFN-**γ**, and IL-1**β** levels but had no effect on the expression of GFAP, TNF-**α**, and COX-2 compared to the cuprizone group. Sildenafil elevated GSTpi levels and improved the myelin structure/ultrastructure. iNOS^−/−^ mice suffered from severe inflammation following treatment with cuprizone, while WT mice had milder inflammation, as found in the previous study. It is possible that inflammatory regulation through iNOS-feedback is absent in iNOS^−/−^ mice, making them more susceptible to inflammation. Sildenafil has at least a partial anti-inflammatory effect through iNOS inhibition, as its effect on iNOS^−/−^ mice was limited. Further studies are required to explain the underlying mechanism of the sildenafil effects.

## 1. Background 

Nitric oxide (NO) is a highly reactive molecule with a range of physiological functions [1, 2]. This messenger plays an important role in the modulation of vascular tone [3], neurotransmission [4, 5], and immune system [6–8]. NO is produced from L-arginine by NO synthases (NOSs). In addition to the constitutive forms of the enzyme (endothelial (eNOS or NOS3) and neuronal (nNOS or NOS1)), there is also an inducible form (iNOS or NOS2). This last is most commonly associated with inflammatory conditions in which NO is produced in large amounts.

There is strong evidence to suggest the involvement of iNOS in the development of neurodegenerative disease. Induction of iNOS, NO, and NO byproducts has been found in multiple sclerosis (MS) patients and animal models and correlates with disease severity and level of inflammatory infiltrate [9–12]. Interestingly, however, iNOS-deficient mice developed a more severe MS model [13, 14]. It was found that the elimination of iNOS does not improve, and may actually aggravate, demyelination in the cuprizone-demyelinating model [15], which suggests that the iNOS/NO system may be neuroprotective. More information is required to understand the role of iNOS/NO in inflammatory response, oligodendrocyte cell death, and myelin damage/loss.

The cyclic guanosine 3′,5′-monophosphate (cGMP) signaling pathway is an important NO-signaling molecule. NO binds to soluble guanylyl cyclase (sGC) and increases concentration of cGMP, activating signaling cascades and leading to cGMP-dependent responses [16, 17]. The cGMP signal can be terminated by the action of several phosphodiesterases (PDE) [18]. The cGMP-selective PDE5 is expressed in the cardiovascular, neural, and immune systems [18]. Studies have shown that selective PDE5 inhibitors, widely used in the treatment of erectile dysfunction in humans, such as sildenafil (Viagra; Pfizer) and vardenafil (Levitra; Bayer), raise cGMP levels in the brain and offer protective effects, improve cognition and memory [19], reduce neuronal cell death following ischemic cerebrovascular injury [20], decrease white matter damage, and regulate inflammatory responses in MS models [21, 22]. Recent studies by the authors of an MS model induced in wild-type C57BL6 mice found that sildenafil has an anti-inflammatory action, reducing levels of proinflammatory cytokines and cyclooxygenase-2 (COX-2) and protecting the myelin structure [22]. However, the mechanism of sildenafil neuroprotection remains unknown. 

In the present study, inflammatory demyelination was induced in iNOS^−/−^ mice, and sildenafil was administered for four weeks. The focus of this study was to identify the role of a potent inflammation-associated molecule, iNOS-derived NO, in protective mechanisms related to sildenafil. The present study also aimed to clarify the role of NO protective and/or deleterious mechanisms in the demyelinating model.

## 2. Materials and Methods

### 2.1. Experimental Design

Five iNOS knockout (B 6.129 P2- Nos2) mice, aged 7 to 10 weeks, weighing 15 to 20 g, were used per group. The mice were examined for health status, acclimated to the laboratory environment at 25°C and 12 h light/dark photoperiod, and housed in metal cages. The control group received standard laboratory diet and pure water. Over a four-week period, the experimental groups received either 0.2% cuprizone (oxalic-bis-cyclohexylidenehydrazide Sigma-Aldrich Inc., St. Louis, MO, USA) mixed into the chow and pure water or 0.2% cuprizone in the chow and 25 mg/kg of body weight of sildenafil (Viagra; Pfizer Inc., New York, NY, USA) administered through the drinking water [22–24]. Body weight was accessed every week and the drug concentration in the water was adjusted to maintain the dose. All experiments were carried out in compliance with ethical guidelines for animal experimentation (L-10/2010-CEUA; 05/10-CIBIO FIOCRUZ). After treatment, the animals were anaesthetized (i.m.) with ketamine (115 mg/kg) and xylazine (10 mg/kg) (Sespo Comércio e Indústria Ltda., São Paulo, SP, Brazil).

### 2.2. Immunofluorescence (IF)

After anesthesia, the animals were transcardially perfused with physiological saline (20 mL), followed by 4% paraformaldehyde (Sigma-Aldrich) (40 mL) in 0.1 M phosphate (sodium phosphate monobasic and dibasic heptahydrate, Sigma-Aldrich) buffered saline (PBS), pH 7.2. Cerebella were dissected and immersed in 15% sucrose overnight, followed by 30% sucrose for a second night (36 hours total). The specimens were then embedded in OCT-Tissue Tek compound (Sakura Finetek, Torrance, CA, USA) and frozen in n-hexane (Dinâmica, São Paulo, SP, Brazil) cooled with liquid nitrogen. Cryosections (8 *μ*m thick) were permeabilized (0.3% Triton X-100) and incubated for 1 h with blocking solution (3% BSA plus 0.2% Tween 20 in Tris buffered saline). Subsequently, the sections were incubated with antibodies for Glial Fibrillar Acidic Protein (GFAP) (DakoCytomation, cat. no. ZO 334) and Iba-1 (Wako, Osaka, Japan, cat. no. 019-19741) (both 1 : 100). Sections were incubated with primary antibodies overnight and then incubated with polyclonal Cy3-conjugated secondary antibodies (Jackson, cat. no. 705-165-147) against rabbit immunoglobulin (1 : 200) for 1 h. The slides were washed and mounted in fluorescent Prolong Gold Antifade medium (Life Technologies, cat. no. P36930) for observation under an inverted fluorescence microscope (Zeiss MicroImaging GmbH) equipped with a camera (Zeiss AxioCam MRM) and the Release 4.7.2 image analysis software.

### 2.3. Immunohistochemistry (IH)

After perfusion as described for IF, cerebella were immediately removed and postfixed in the same fixative overnight. The samples were dehydrated in an ethanol series (Isofar Chemical Co., RJ, Brazil), cleared in xylene, and embedded in paraffin (Merck, catalog no. 1071642504). Sections (5 *μ*m thick) were cut on an RM 2035 microtome (Reichert S, Leica), rehydrated, washed in 0.05 M PBS, and incubated in this buffer with 1% bovine serum albumin (BSA, fraction V) (Miles, Naperville, IL, USA) for one hour. Endogenous peroxidase was blocked and antigen retrieval was performed, pretreating the sections with 20 mM citrate buffer, pH 6.0, at 100°C, for 30 min. All groups were incubated with the rabbit polyclonal anti-COX-2 (Abcam, Canada/US, cat. no. ab15191) (1 : 100, overnight at 4°C). After washing, the sections were overlaid for 1 h with a biotin-conjugated secondary antibody using an HRP kit (DakoCytomation, CA, USA, Biotinylated Link Universal HRP; cat. no. K0690) and visualized with 3′-3-diaminobenzidine (DAB) as the chromogen. The sections were then weakly counterstained with Harris' hematoxylin and mounted in entellan (Merck, cat. no. 1079610100).

### 2.4. Western Blotting (WB)

Cerebella were quickly dissected, and each group was homogenized in an extraction cocktail (10 mM EDTA, Amresco, Solon, USA; 2 mM phenylmethane sulfonyl-fluoride, 100 mM NaF, 10 mM sodium pyrophosphate, 10 mM NaVO_4_, 10 *μ*g of aprotinin/mL, and 100 mM Tris, pH 7.4). Cerebella of five animals were mixed and homogenized to form a pool from each group. The WB was done in accord with Nunes et al. [22]. Briefly, the proteins (40 *μ*g total) were separated on 6.5% (IFN-*γ*), 10% (TNF-*α*, IL-1*β*), or 12% (GFAP, eNOS) acrylamide gel. After overnight in 5% nonfat milk, the primary antibodies against GFAP (1 : 10,000, DakoCytomation, cat. no. ZO 334), COX-2 (1 : 1000, Abcam, cat. no. ab15191), TNF-*α* (1 : 1000, Peprotech, NJ, USA, cat. no. 500-P64), IFN-*γ* (1 : 2500, Peprotech cat. no. 500P119), IL-1*β* (1 : 2500, GenWay Biotech, Inc., CA/USA, cat. no. 18-732-292194), GST3/GSTpi (1 : 250, Abcam, Canada, cat. no. ab53943), and eNOS (1 : 1000, BD Biosciences, cat. no. 610299) were incubated for four hours followed by horseradish peroxidase-conjugated antibodies anti-rabbit (1 : 80,000, Sigma-Aldrich, cat. no. A9169), anti-mouse (1 : 1,000, Sigma-Aldrich, cat. no. A0168), or anti-goat secondary antibody (1 : 100,000, Sigma-Aldrich, cat. no. A5420). For quantification, the pixel density of each band was determined using the Image J 1.38 software (http://rsbweb.nih.gov/ij/download.html; developed by Wayne Rasband, NIH, Bethesda, MD, USA). For each protein investigated, the results were confirmed in three sets of experiments. Immunoblotting for *β*-actin (1 : 1,000, Sigma-Aldrich, cat. no. A2228) was performed as a control.

### 2.5. Luxol Fast-Blue (LFB)

After perfusion as described for IF, the samples were dehydrated in a graded ethanol series, cleared in xylene, and embedded in paraffin. Sections (5 *μ*m thick) were cut on an RM 2035 microtome (Reichert S, Leica). Myelin was detected using Luxol-Fast Blue (LFB) staining (Solvent Blue 38; Sigma-Aldrich) in accord with Nunes et al. [22]. The sections were observed under an inverted microscope (Zeiss MicroImaging GmbH) equipped with a camera (Zeiss AxioCam MRM) and Release 4.7.2 image analysis software (Zeiss).

### 2.6. Transmission Electron Microscopy (TEM)

After anesthesia, the animals were sacrificed by transcardial perfusion with physiological saline (20 mL), followed by 40 mL of fixative—2.5% glutaraldehyde and 4% paraformaldehyde in 0.1 M sodium cacodylate acid (Sigma-Aldrich) buffer, pH 7.2. Cerebella were quickly dissected and postfixed in the same fixative overnight. Next, cerebellum fragments were washed twice in the same buffer and postfixed in a solution containing 1% osmium tetroxide (Sigma-Aldrich), 2 mM calcium chloride, and 0.8% potassium ferricyanide (Sigma-Aldrich) in 0.1 M cacodylate buffer, pH 7.2, dehydrated in acetone and embedded in SPIN-PON resin (Embed 812-Electron Microscopy Science, Washington, PA, USA). Resin polymerization was performed at 60°C for 3 days. Semithin sections (0.5 *μ*m in thickness) were placed on glass slides, stained with toluidine blue. Ultrathin sections (70 nm in thickness) were placed on 300-mesh nickel grids, counterstained with 5% uranyl acetate (Electron Microscopy Science) and lead citrate (Sigma-Aldrich), and examined using a FEI Morgagni 268D transmission electron microscope. 

### 2.7. Statistical Analysis

The densitometric values of the immunoreactive bands (immunoblotting) were analyzed using the GraphPad Prism software package (San Diego, CA, USA). One-way analysis of variance (ANOVA), followed by Dunnett's and/or Tukey's posttest, was used to compare groups. The results were expressed as means ± SE, when appropriate. A *P* value < 0.05 indicated statistical significance.

## 3. Results

Clinical analysis revealed that cuprizone-treated animals suffered from motor limitations, such as tremors, and abnormal walking and posture. The group treated with sildenafil (25 mg/kg) exhibited normal walking and posture, and tremors were either mild or nonexistent.

The clinical signs were observed and recorded by three observers. The iNOS^−/−^ control animals exhibited normal motor function and posture and explored their environment normally. This group was classified as score 0 (no sign). The mice treated with cuprizone exhibited arched (shortened) posture and tremors and had difficulty in exploring the environment. This group was classified as score 2. Mice treated with 25 mg/kg of sildenafil both walked and were able to explore the environment normally, with no or mild tremors, and were classified as score 1.

Cuprizone increased GFAP, TNF-*α*, and COX-2 expression in cerebellum, indicating astrocyte activation (reactive gliosis) and neuroinflammation. Sildenafil treatment did not reduce the levels of these proteins in mice without iNOS.

**Figure 1:**
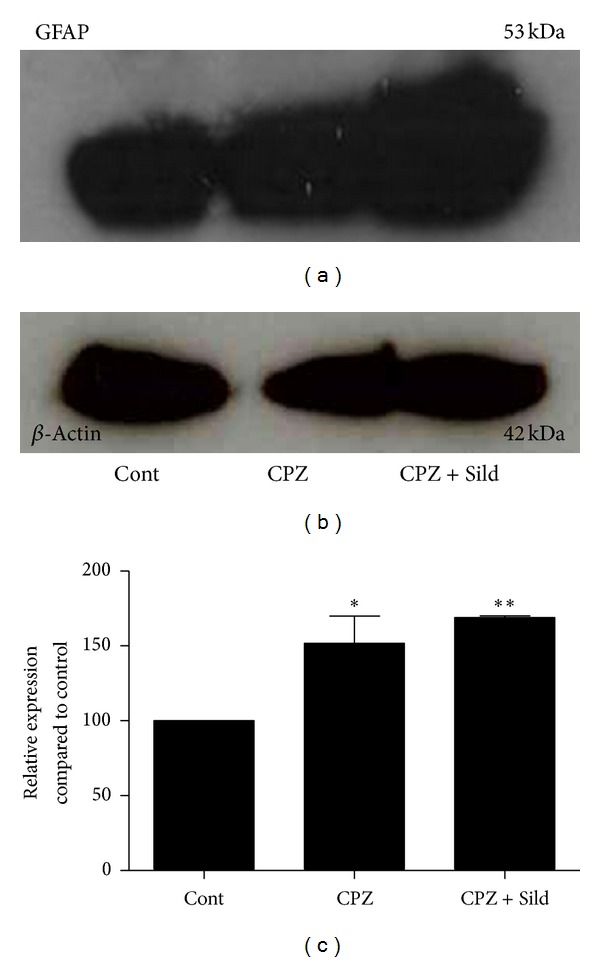
Western blotting for GFAP. (a) GFAP immunoblot of iNOS^−/−^ control (Cont), cuprizone (CPZ), and cuprizone plus sildenafil (CPZ + Sild) groups. (b) *β*-actin immunoblot. (c) Graph represents quantification and statistical analysis. The control group showed basal expression of GFAP. CPZ treatment induced a significant increase of this protein and sildenafil plus CPZ did not reduce GFAP expression, which remained higher in relation to the control group. The experiment was performed in triplicate (*n* = 5 animals/group). The results were expressed as mean ± SE. *P < 0.05, **P < 0.01 compared to control.

 Western Blotting (WB) analysis showed that GFAP, a marker of astrocyte activation (reactive gliosis), was present in the cerebellum of untreated iNOS^−/−^ animals (control) (Figure 1(a)). Treatment with 0.2% cuprizone for four weeks significantly increased expression of this protein (Figures 1(a) and 1(c); *P* < 0.001). Animals that concomitantly received sildenafil (25 mg/Kg) and cuprizone also exhibited a high level of GFAP in comparison to control (Figures 1(a) and 1(c); *P* < 0.001).

**Figure 2:**
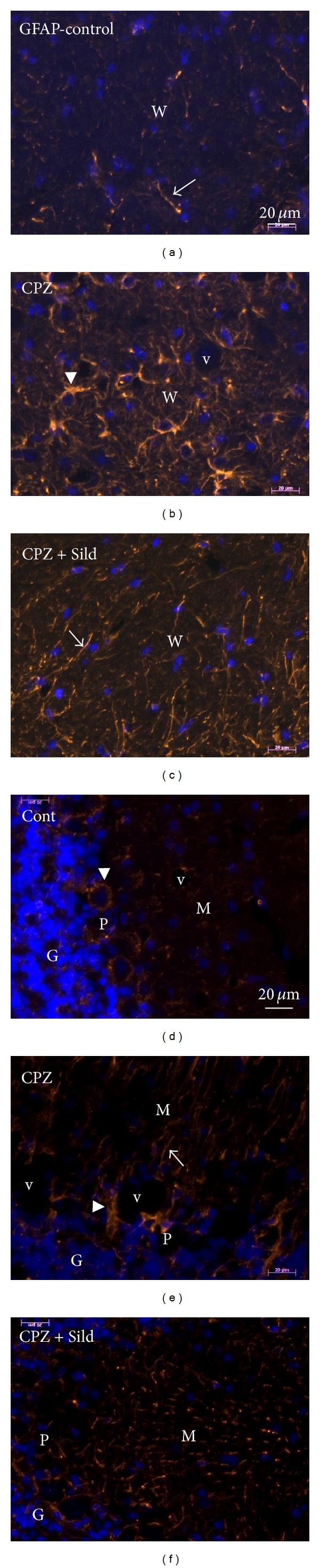
Immunofluorescence for GFAP. (a) and (d) show expression and physiological locations of GFAP in mice without iNOS and without treatment. CPZ administration ((b), (e)) induced reactive gliosis, with thicker and more numerous astrocytic processes, compared to control. GFAP remained high in relation to control, after application of sildenafil plus CPZ ((c), (f)). Arrows show astrocytic processes and arrowheads point to processes around vessels and other cells. W: white matter, M: molecular layer, P: purkinje layer, G: granular layer, and v: vessel. Bars: 20 *μ*m.

Immunofluorescence (IF) analysis of GFAP in the cerebellum revealed the expression and location of this cytoskeletal intermediate filament protein in the animals. In the molecular layer of the cerebellum, GFAP labeling revealed long astrocytic processes with a typical arrangement that was perpendicular to the pia mater membrane (Bergmann glia) (Figure 2(e)). Astrocytic processes were also seen around other cells and vessels (arrowheads in Figures 2(b), 2(d), and 2(e)). The control group showed basal expression of GFAP localized normally (Figures 2(a) and 2(d)). Treatment with CPZ induced astrogliosis, increasing the intensity of labeling in the astrocytes (Figures 2(b) and 2(e)). In the sildenafil group, GFAP labeling was more intense than for control (Figures 2(c) and 2(f)).

**Figure 3:**
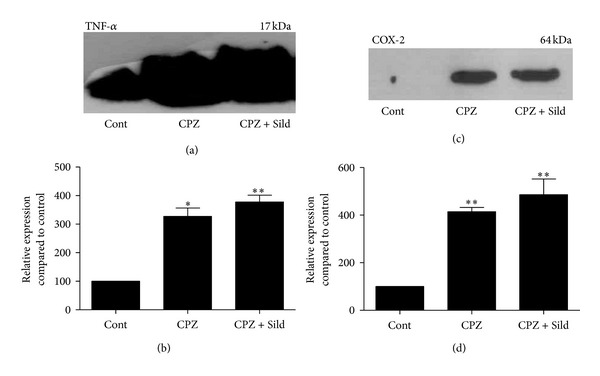
Western blotting for TNF-*α* and COX-2. ((a), (c)) Immunoblots of iNOS^−/−^ control (Cont), cuprizone (CPZ), and cuprizone plus sildenafil (CPZ + Sild) groups. ((b), (d)) Graphs represent quantification and statistical analysis. The control group showed basal expression of TNF-*α*. CPZ treatment caused a significant increase of this cytokine, and sildenafil plus CPZ did not decrease its expression, which remained higher in relation to the control group. Only minimum amounts of COX-2 were present in the control group. CPZ and CPZ + Sild caused a significant increase of this enzyme, compared to control. The experiment was performed in triplicate (*n* = 5 animals/group). The results were expressed as mean ± SE. *P < 0.05, **P < 0.01 compared to control.

The iNOS^−/−^ control group had a basal level of TNF-*α* (Figure 3(a)). WB analysis showed that cuprizone induced a significant increase of this cytokine, indicating neuroinflammation (Figures 3(a) and 3(b); *P* < 0.05, compared to control group). Animals that received cuprizone plus sildenafil also had a significant increase of TNF-*α*, compared to control (*P* < 0.01). 

**Figure 4:**
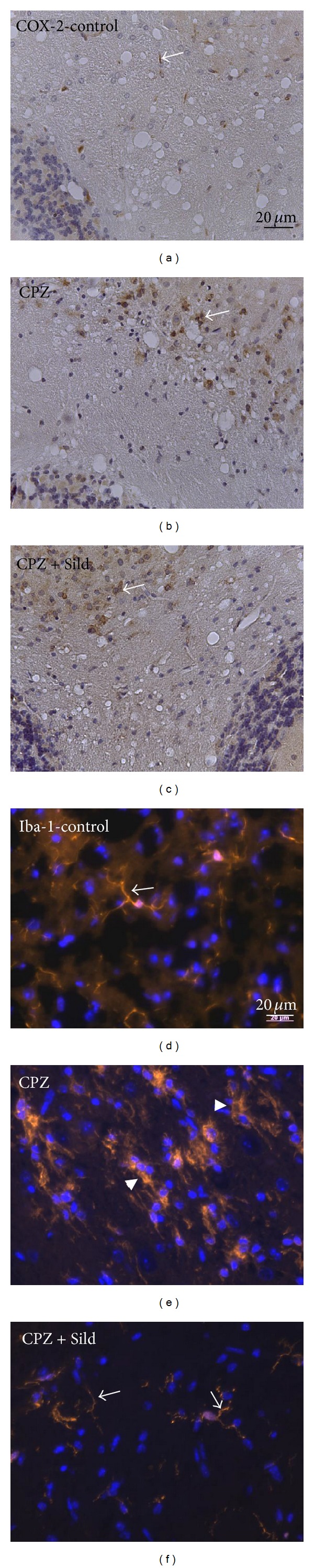
Immunohistochemistry for COX-2 ((a)–(c)) and immunofluorescence for Iba-1 ((d)–(f)). iNOS^−/−^ control (a) showed very low COX-2 expression (arrow). After CPZ treatment (b), COX-2 labeling significantly increased, mainly in white matter, in relation to control. Animals treated with CPZ + Sild (c) also increased COX-2, comparing to the control group. A basal expression of Iba-1 was seen in control animals without iNOS (d). CPZ increased Iba-1 and induced an activated phenotype (arrowheads) of microglia (e). Sildenafil plus CPZ decreased Iba-1 and induced latent phenotype (arrows) of microglia (f).

COX-2 was analyzed by WB and immunohistochemistry (IH) tests. This enzyme was expressed in a minimal amount in the cerebellum of the iNOS^−/−^ control group (Figures 3(c) and 4(a)). CPZ administration induced a significant increase in COX-2 expression, compared to control (Figures 3(c) and 3(d); *P* < 0.01). Sildenafil treatment did not decrease COX-2, which remained high in comparison with the control group (Figures 3(c) and 3(d); *P* < 0.01). IH labeling revealed the expression and location of COX-2 (Figures 4(a)–4(c)), which was mainly found in the white matter of the cerebellum. The control group had a very low expression of this enzyme (Figure 4(a)), while treated animals had a high expression of COX-2 in white matter (Figures 4(b) and 4(c)). 

Immunoblot control with *β*-actin is shown in Figure 1(b).

Cuprizone increased Iba-1, IL-1*β*, and IFN-*γ* expression in the cerebellum of iNOS^−/−^ mice. While sildenafil decreased the expression of these proteins, the level of expression remained above that of control animals.

 The microglial marker, Iba-1, was analyzed by IF. There was physiological expression of Iba-1 in the cerebellum of iNOS^−/−^ control animals, with cells with processes that were typically branched, thin and weakly labeled (arrow, Figure 4(d)). Cuprizone treatment induced a stronger expression of Iba-1 (Figure 4(e)), compared to the control group, with thicker and more intensely labeled microglial processes. These processes lost their typical thin branched appearance, indicating that the cell phenotype had acquired activated characteristics. Sildenafil together with CPZ decreased Iba-1 expression, and the microglia exhibited thin, highly branched processes, typical of a latent state (Figure 4(f)). 

**Figure 5:**
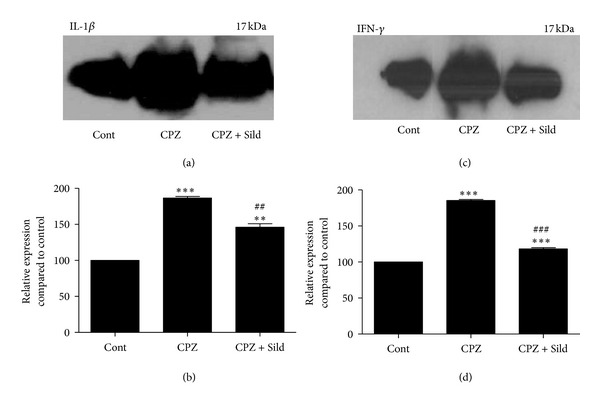
Western blotting for IL-1*β* and IFN-*γ*. ((a), (c)) Immunoblots of iNOS^−/−^ control (Cont), cuprizone (CPZ), and cuprizone plus sildenafil (CPZ + Sild) groups. ((b), (d)) Graphs represent quantification and statistical analysis. The control group showed basal expression of IL-1*β* and IFN-*γ*. CPZ treatment induced a significant increase of these cytokines, compared to control. Sildenafil plus CPZ significantly decreased IL-1*β* and IFN-*γ* expression, in relation to the CPZ group. The experiment was performed in triplicate. The results were expressed as mean ± SE. **P < 0.01, ***P < 0.001, compared to control; ^##^ *P* < 0.01, ^###^ *P* < 0.001, compared with CPZ.

Immunoblotting for IL-1*β* and IFN-*γ* revealed basal expression of these cytokines in the iNOS^−/−^ control group (Figures 5(a) and 5(c)). Cuprizone strongly increased the expression of these cytokines (both *P* < 0.001), compared to the control group (Figures 5(a)–5(d)), which indicates neuroinflammation. Sildenafil treatment resulted in a significant decrease of IL-1*β* and IFN-*γ* compared to the cuprizone group (*P* < 0.01). However, the levels of IL-1*β* and IFN-*γ* remained higher in the sildenafil-treated group when compared with baseline levels of the control group (*P* < 0.01 and *P* < 0.001, resp.).

Mice without iNOS underwent a significant increase in eNOS expression, compared to wild-type animals.

**Figure 6:**
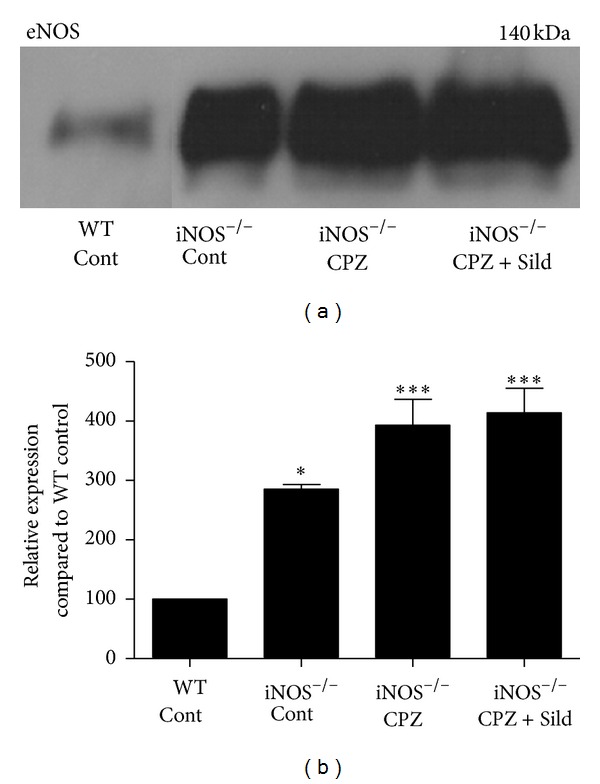
Western blotting for eNOS. (a) Immunoblot of wild-type mice control (WT cont), iNOS^−/−^ mice control (iNOS^−/−^ cont), iNOS^−/−^ animals treated with cuprizone (iNOS^−/−^ CPZ), and iNOS^−/−^ animals treated with cuprizone plus sildenafil (iNOS^−/−^ CPZ + Sild). (b) Graph represents quantification and statistical analysis. eNOS was physiologically expressed in WT mice. Animals without iNOS without treatment showed a significant increase of this enzyme, compared to WT control. After CPZ and CPZ plus sildenafil, iNOS^−/−^ animals also showed a significant increase of eNOS, compared to WT animals, but no significant difference was identified between iNOS^−/−^ control and treated animals. The experiment was performed in triplicate. The results were expressed as mean ± SE. *P < 0.05, ***P < 0.001, compared to control.

 Untreated mice iNOS^−/−^ (control group; *P* < 0.01) and iNOS^−/−^ animals treated with CPZ or CPZ + Sild (both *P* < 0.001) showed a significant increase in eNOS levels, compared with wild-type mice that did not undergo treatment. The expression of eNOS in iNOS^−/−^ mice treated with CPZ and CPZ plus sildenafil was not significantly higher when compared to iNOS^−/−^ control animals but showed a propensity to increase (Figures 6(a) and 6(b)). 

Cuprizone decreased GSTpi, indicating the depletion of mature oligodendrocytes. Sildenafil increased GSTpi expression in the cerebellum of iNOS^−/−^ mice.

**Figure 7:**
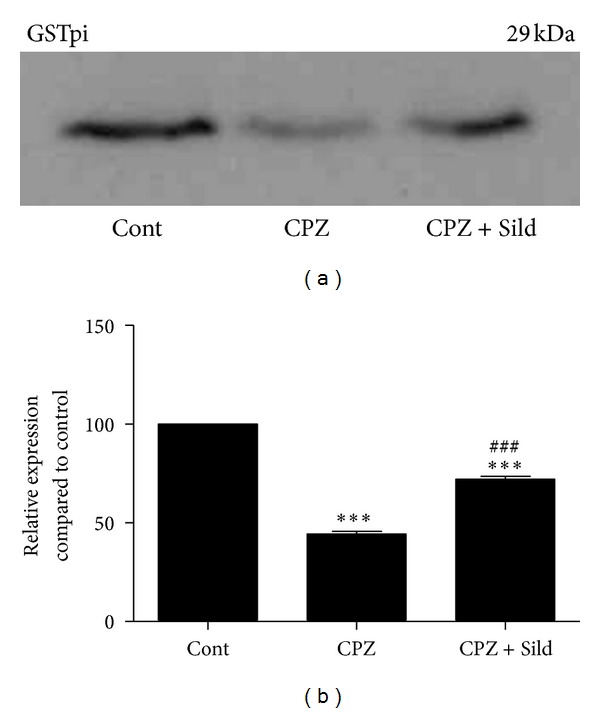
Western blotting for GSTpi. (a) Immunoblot of iNOS^−/−^ control (Cont), cuprizone (CPZ), and cuprizone plus sildenafil (CPZ + Sild) groups. (b) Graph represents quantification and statistical analysis. iNOS^−/−^ control showed basal expression of GSTpi. After CPZ, GSTpi decreased significantly, compared to iNOS^−/−^ cont. CPZ + Sild treatment increased GSTpi expression in relation to the CPZ group, but the levels of this protein remained significantly decreased compared to control. The experiment was performed in triplicate. ***P < 0.001 compared to control; ^###^ *P* < 0.001 compared to CPZ.

 GSTpi, a marker of myelinating oligodendrocytes, was expressed in the cerebellum of the control group (Figure 7(a)). After treatment with CPZ, this marker decreased significantly (*P* < 0.001) compared to the control group, suggesting an impairment of mature oligodendrocytes and consequent myelin damage. Sildenafil treatment together with CPZ induced partial recovery of the GSTpi marker, indicating that sildenafil had a protective effect on mature oligodendrocytes. In the sildenafil group, GSTpi decreased in comparison with control (*P* < 0.001) but increased in comparison to the cuprizone group (*P* < 0.001; Figures 7(a) and 7(b)).

Myelin sheath structure was disorganized in animals without iNOS which did not undergo treatment (control). Cuprizone induced more severe myelin disruption. Sildenafil improved myelin structure and ultrastructure.

**Figure 8:**
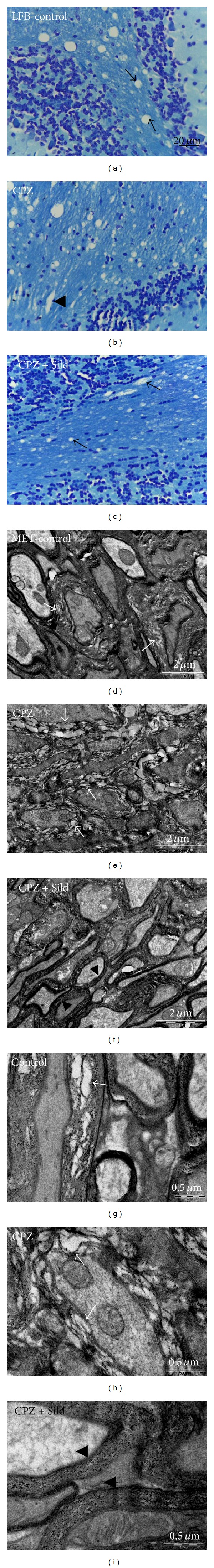
Luxol Fast Blue (LFB) staining ((a)–(c)) and electron micrographs ((d)–(i)). (a), (d), and (g) represent iNOS^−/−^ control group; (b), (e), and (h) represent CPZ-treated animals; (c), (f), and (i) represent CPZ + Sild-treated animals. Arrows in (a), (c): vacuoles in the white matter; arrowhead in (b): spaces between fibers; arrows in (d), (e), (g), and (h): damaged myelin sheath; arrowheads in (f), (i): preserved myelin. Bars = 20 *μ*m ((a)–(c)); 2 *μ*m ((d)–(f)); 0.5 *μ*m ((g)–(i)).

 Interestingly, iNOS^−/−^ animals without treatment (control) had moderate disorganization of the myelin structure and ultrastructure (Figures 8(a), 8(d), and 8(g)). Standard LFB staining showed that the control group iNOS^−/−^ had vacuoles in white matter (arrow in Figure 8(a)), resulting in inhomogeneous and disorganized tissue. Qualitative analysis of ultrathin cerebellum sections by TEM revealed that the myelin sheath was often shredded, with blanks, and without the characteristic lamellar pattern (arrows in Figures 8(d) and 8(g)). 

Cuprizone-treated animals exhibited more severe damage to myelin structure and ultrastructure (Figures 8(b), 8(e), and 8(h)). LFB staining (Figure 8(b)) showed vacuoles (arrows) and spaces (arrowhead) as slits in white matter, characteristic of highly disorganized tissue. TEM revealed that CPZ caused serious damage to the myelin sheath ultrastructure, which had numerous spaces between the shreds in practically all fibers (represented by arrows in Figures 8(e) and 8(h)). The typical lamellar pattern was entirely absent. 

Simultaneous treatment with sildenafil and CPZ resulted in a noticeable improvement of myelin organization (Figures 8(c), 8(f), and 8(i)). White matter was more homogeneous, presenting fewer and, in general, smaller vacuoles and slit-like spaces (Figure 8(c)). Ultrathin section analysis revealed a more preserved myelin sheath which rarely showed signs of shredding. 

## 4. Discussion

 The cuprizone model is characterized by primary and reversible demyelination, due to peripheral immune system-independent myelin injury [25]. In this model, demyelination is accompanied by a well-characterized sequence of events involving the depletion of mature oligodendrocytes, microglia activation, and astrocyte proliferation [26]. Therefore, demyelination and resident neuroinflammation induced by cuprizone in rodents have been widely used as a model for MS [20, 27]. In the present study, the usefulness of the cuprizone model is considerable as it allows evaluation of the role of the iNOS/NO-sGC-cGMP pathway in the inflammation and demyelination mediated by resident CNS cells. 

 The cerebellum was chosen for analysis, as it is an important CNS affected region in MS patients, revealing severe white matter atrophy [28, 29]. Furthermore, the authors of the present study had recently investigated this region [22]. The present data, relating to iNOS^−/−^ animals, will be discussed with reference to this previous study. Nunes et al. (2012) [22] showed that in C57BL/6 wild-type (WT) mice cuprizone induced tissue damage, increased GFAP, Iba-1, and COX-2 expression, and caused demyelination, in comparison to the control group. However, cuprizone did not affect the expression of cytokines (TNF-*α*, IFN-*γ*, IL-1*β*, and IL-2) in WT mice. In the previous study by the authors, sildenafil reduced GFAP and Iba-1 expression in comparison to the cuprizone group, preserved myelin and axon ultrastructure, and significantly downregulated IFN-*γ*, TNF-*α*, IL-1*β*, IL-2, and COX-2 expression in comparison to the control and/or cuprizone groups. The previous results demonstrated the protective effect of sildenafil on the cerebellum. Sildenafil promotes the accumulation of cGMP, which is the main NO signaling molecule. It was considered important to investigate the role of NO in the MS-model and the effects of sildenafil more profoundly. Therefore, in the present study, the relationship between iNOSnull mice, the MS-model, and the effects of sildenafil in the cerebellum was investigated. 

 In the absence of iNOS/NO, cuprizone significantly increased expression of GFAP, TNF-*α*, COX-2, Iba-1, IL-1*β*, and IFN-*γ*. In addition, cuprizone intoxication decreased GSTpi, a marker for myelinating oligodendrocytes, damaged myelin, and induced tremors, abnormal walking, and posture. This data indicates that cuprizone intoxication occurred even without the iNOS-NO system and was stronger than in WT mice, where cuprizone did not increase cytokine expression [22]. Interestingly, iNOS^−/−^ control animals showed an altered myelin structure. 

 It was hypothesized that, in the absence of iNOS, eNOS may be overexpressed as a compensatory mechanism. Bernardini et al. [30] showed that treatment with endotoxin influenced NOS expression, upregulating iNOS and, simultaneously, downregulating eNOS. There appeared to be a regulatory relationship between the expression of iNOS and eNOS. In fact, it was found here that eNOS was strongly expressed in iNOS^−/−^ animals in comparison with WT mice. The eNOS levels remained high after the administration of cuprizone and cuprizone plus sildenafil. In normal conditions, low levels of NO produced by both eNOS and nNOS participate in cell signaling and regulate physiologic processes [31]. However, eNOS overexpression (and consequently, constant high concentration of NO) can be responsible for myelin changes and proinflammatory susceptibility in iNOS^−/−^ animals. 

 On the other hand, iNOS possesses an important feedback mechanism in inflammatory conditions, when the increase of this enzyme is self-regulated, and induces a reduction of some proinflammatory proteins [32–35]. The inhibition of iNOS activity induces enhancement of IL-1*β*, IL-6, and TNF-*α* levels [36] and, subsequently, a persistent increase of iNOS expression, downregulating the TNF receptor [35]. In the present study, absence of iNOS may explain increased TNF-*α*, IFN-*γ*, and IL-1 after cuprizone treatment in iNOS^−/−^ mice, while cuprizone did not increase these cytokines in WT mice [22]. This feedback mechanism is coregulated by a high concentration of cGMP [32]. Therefore, another hypothesis for explaining the more severe inflammation induced by cuprizone in mice without iNOS is the absence of iNOS feedback mechanism. 

 Interestingly, although sildenafil had a low anti-inflammatory effect on iNOS^−/−^ mice, it considerably improved the myelin structure of mice without iNOS. Cuprizone is a copper chelator which leads to direct oligodendrocyte death with subsequent demyelination [37]. In this model, oligodendrocyte death and demyelination are independent of immune and inflammatory response. It was found that cGMP analog (8-Br-cGMP) protects differentiated oligodendrocytes from death initiated by staurosporine, thapsigargin, or kainate [38]. It is possible that sildenafil, through the accumulation of cGMP, has a direct beneficial effect on oligodendrocytes, protecting these cells and improving myelination, independent of its anti-inflammatory effects. 

 In conclusion, the findings of the present study show that iNOS^−/−^ mice are more susceptible to cuprizone intoxication due to the potential involvement of two mechanisms: (1) iNOS-negative feedback mechanism in inflammatory conditions is absent and, consequently, proinflammatory proteins, such as cytokines and COX-2, are excessively increased; (2) eNOS is overexpressed by a compensatory mechanism and generates chronically high levels of NO, damaging the tissue. Also, the results of the present study suggest that sildenafil may exert its anti-inflammatory effects mainly through iNOS inhibition, by cGMP-iNOS feedback. In addition, sildenafil may have a direct protective effect on oligodendrocytes. Further studies are required to explain the molecular mechanism of sildenafil protection in the central nervous system.
